# A systematic review and meta-analysis of percutaneous coronary intervention compared to coronary artery bypass grafting in non-ST-elevation acute coronary syndrome

**DOI:** 10.1038/s41598-022-09158-0

**Published:** 2022-03-24

**Authors:** Hristo Kirov, Tulio Caldonazo, Mohamed Rahouma, N. Bryce Robinson, Michelle Demetres, Patrick W. Serruys, Giuseppe Biondi-Zoccai, Mario Gaudino, Torsten Doenst

**Affiliations:** 1 Department of Cardiothoracic Surgery, Friedrich-Schiller-University Jena, 101 Erlanger Allee, 07747 Jena, Germany; 2 Department of Cardiothoracic Surgery at New York Presbyterian, Weill Cornell Medical Center, New York, USA; 3 Samuel J. Wood Library and C.V. Starr Biomedical Information Center, Weill Cornell Medicine, New York, NY USA; 4 Department of Cardiology, National University of Ireland, Galway (NUIG), Galway, Ireland; 5 CORRIB Corelab and Center for Research and Imaging, Galway, Ireland; 6 NHLI, Imperial College London, London, UK; 7 Department of Medico-Surgical Sciences and Biotechnologies, Sapienza University of Rome, Latina, Italy; 8 Mediterranea Cardiocentro, Naples, Italy

**Keywords:** Cardiovascular biology, Interventional cardiology

## Abstract

Non-ST-elevation acute coronary syndrome (NSTE-ACS) affects millions of patients. Although an invasive strategy can improve survival, the optimal treatment [i.e., percutaneous coronary intervention (PCI) or coronary artery bypass grafting (CABG)] is not clear. We performed a meta-analysis of studies reporting outcomes between PCI and CABG in patients with NSTE-ACS. MEDLINE, EMBASE and Cochrane Library were assessed. The primary outcome was long-term mortality. Inverse variance method and random model were performed. We identified 13 observational studies (48,891 patients). No significant difference was found in the primary endpoint [CABG vs. PCI, incidence rate ratio (IRR) 0.93, 95% confidence interval (CI) 0.70; 1.23]. CABG was associated with lower long-term major adverse cardiovascular events (MACE) (IRR 0.64, 95% CI 0.54; 0.76) and lower long-term re-revascularization (IRR 0.37, 95% CI 0.30; 0.47). There was no significant difference in long-term myocardial infarction (CABG vs. PCI, IRR 0.96, 95% CI 0.50; 1.84) and peri-operative mortality (CABG vs. PCI, odds ratio 1.36, 95% CI 0.94; 1.95). For the treatment of NSTE-ACS, CABG and PCI are associated with similar rates of long-term mortality and myocardial infarction. CABG is associated with lower rates of long-term MACE and re-revascularization. Randomized comparisons in this setting are necessary.

## Introduction

Non-ST-elevation acute coronary syndrome (NSTE-ACS) represents a large proportion of patients with acute coronary syndromes (ACS) impacting millions of patients worldwide^1,2^. Recently, the percentage of patients presenting with non-ST-elevation myocardial infarction (NSTEMI) has increased in both Europe and United States^1,2^. Although a routine invasive strategy in NSTE-ACS may improve long-term survival and reduce late myocardial infarction^3^, the optimal treatment method remains controversial. Specifically, it remains unclear as to whether percutaneous coronary intervention (PCI) or coronary artery bypass grafting (CABG) provide better long-term outcomes^4^. A possible difference in treatment outcomes might be plausible, as recent publications have shown that PCI and CABG mechanisms differ^5,6^. Although both stents and bypass grafts provide revascularization to vascular territories affected by flow-limiting stenoses, only CABG also provides protection against vessel occlusions from non–flow-limiting stenoses, because the majority of bypass graft insertions are performed distal to plaque location^5,6^.

To date, there is no randomized comparison of PCI vs. CABG surgery in the specific setting of NSTE-ACS. While there have been a number of non-randomized studies published, their results have been inconclusive. Additionally, no systematic review and meta-analysis addressing this topic exists in the literature.

In this analysis we set out to systematically review the literature on the impact of the treatment modality on clinical outcome in NSTE-ACS.

## Methods

This analysis was prospectively registered on the International Prospective Register of Systematic Reviews in Health and Social Care (PROSPERO, ID number CRD42020214423). Ethical and internal review board approval was not required for this analysis as no human or animal subjects were involved.

### Search strategy

A medical librarian (MD) performed a comprehensive literature search to identify contemporary studies comparing outcomes in patients with NSTE-ACS (unstable angina pectoris [UAP] and non-ST-elevation myocardial infarction [NSTEMI]) who underwent either PCI or CABG. Searches were run on October 19, 2020 in the following databases: Ovid MEDLINE® (ALL; 2008 to present); Ovid EMBASE (1974 to present); and The Cochrane Library (Wiley). The search strategy for Ovid MEDLINE is available in Supplementary Table 1.

### Study selection and eligibility criteria

The study selection followed the Preferred Reporting Items for Systematic Reviews and Meta-Analyses (PRISMA) strategy^7^. After de-duplication, records were screened by two independent reviewers (TC and HK). Any discrepancies and disagreements were resolved by a third author (TD). All titles and abstracts were reviewed against pre-defined inclusion and exclusion criteria. Studies were considered for inclusion if they were written in English, reported direct comparison between PCI patients and CABG patients and had at least 1 outcome of interest reported. Studies evaluating refractory angina, chronic coronary disease, conference abstracts, proceedings and case reports were excluded. The safety of the comparability of CABG and PCI in a setting of patients with acute ischemic injury and even heart failure has been previously demonstrated^8^.

Following the first round of screening, full text was pulled for selected studies for a second round of eligibility screening. Reference lists for articles in these selected studies were also searched for any relevant articles not captured by the original search strategy.

### Data abstraction and quality assessment

The data extraction and the quality assessment were performed independently by two different investigators (TC and HK) and verified by a third investigator (TD) for accuracy. The following variables were extracted: age, sex, left ventricular ejection fraction, hypertension, diabetes, smoking status, prior cerebrovascular accident, prior myocardial infarction, prior percutaneous coronary intervention, number of vessels addressed (1, 2 or 3), use of drug eluting stents, left main disease and discharge with ASA and/or ADP-inhibitor. Risk of bias was assessed based on Newcastle–Ottawa assessment scale (Supplementary Table 2)^9^.

### Outcomes and effect summary

The primary outcome was long-term mortality (> 6 months). Mean follow up by study is summarized in Table 1. Secondary outcomes were long-term major adverse cardiovascular events (defined in Supplementary Table 3), long-term re-revascularization, long-term myocardial infarction, peri-operative mortality (in-hospital and 30-day events) and long-term stroke. For the primary outcome, a sensitivity analysis comparing adjusted and low risk of bias studies vs. unadjusted studies was also performed.

**Table 1:** Studies included in the meta-analysis.

Study	Study design	Demographics comparability	Country	Patients syndrom included	No patients	Endpoints included	Mean follow-up (Y)
De Feyter^10^	Prespecified analysis—arts trial	Unadjusted	Netherlands	Unstable angina	450	MORTl, MACE, MI, RR	1
Chew^11^	Post-hoc analysis—synergy trial	Unadjusted	Australia	Unstable angina and NSTEMI	9902	MORTl, MI	0.5
Hochholzer^12^	Prospective cohort study	Multivariable regression	Germany	Unstable angina and NSTEMI	1024	MORTl, MI	1.3
Alhabib^13^	Prospective study—gulf race-2 registry	Unadjusted	Saudi Arabia	Unstable angina and NSTEMI	802	MORTp, MORTl	1
Roe^14^	Retrospective—crusade registry	Unadjusted	United States	NSTEMI	15,281	MORTl, MACE, RR	3.2
Buszman^15^	Prospective study—milestone registry	Propensity score matching	Poland	Unstable angina and NSTEMI	4566	MORTp, MORTl	3
Ben-Gal^16^	Post-hoc analysis—acuity trial	Propensity score matching	Israel	Unstable angina and NSTEMI	1772	MORTp, MORTl, MACE, MI, RR	1
Kurlansky^17^	Retrospective—care registry	Propensity score matching	United States	NSTEMI	3228	MACE	5.6 (CABG) AND 5.1 (PCI)
Chang^18^	Patient level data—best/precombat/syntax	Unadjusted	South Korea	Unstable angina and NSTEMI	1246	MORTl, MACE, MI, RR	5
Freitas^19^	Prospective observational study	Multivariable regression	Portugal	NSTEMI	688	MORTl	4.8
Huckaby^20^	Retrospective	Multivariable regression	United States	NSTEMI	2001	MORTl, MACE, MI, RR	3.6
Jia^21^	Prospective observational study	Multivariable regression	China	Unstable angina and NSTEMI	2819	MORTl, MACE, MI, RR	7.5
Ram^22^	Prospective study –acsis registry	Multivariable regression	Israel	Unstable angina and NSTEMI	5112	MORTp, MORTl	3

*CABG* coronary artery bypass grafting, *MACE* major adverse cardiovascular events, *MI* myocardial infarction, *MORTl* long-term mortality, *MORTp* peri-operative mortality, *NSTEMI* non-ST-elevation myocardial infarction, *PCI* percutaneous coronary intervention, *RR* re-revascularization.

### Data analysis

Peri-operative binary outcomes were reported as odds ratios (OR) while long-term outcomes, were reported as incidence rate ratio (IRR); for both estimates the generic inverse variance method was used and 95% confidence intervals (CIs) were also presented.

Random effect meta-analysis was performed using “metafor” and “meta” package^23,24^. CABG was the reference for all pairwise comparisons. Heterogeneity was reported as low (I^2^ = 0–25%), moderate (I^2^ = 26–50%), or high (I^2^ > 50%)^25^. Leave-one-out analysis for the primary outcome was performed to assess the robustness of the obtained estimate. Meta-regression was used to explore the effects of: year of publication, mean follow-up time, age, sex, left ventricular ejection fraction, hypertension, diabetes, smoking status, prior cerebrovascular accident, prior myocardial infarction, prior percutaneous coronary intervention, number of vessels addressed (1, 2 or 3), use of drug eluting stents, left main disease and discharge with ASA and/or ADP-inhibitor on the IRR of the primary outcome.

Statistical significance was set at the 2-tailed 0.05 level, without multiplicity adjustments. All statistical analyses were performed using R (version 3.3.3, R Project for Statistical Computing) within RStudio.

### Ethic declaration

Ethical and internal review board approval was not required for this analysis as no human or animal subjects were involved.

## Results

### Description of included studies

**Figure 1:**
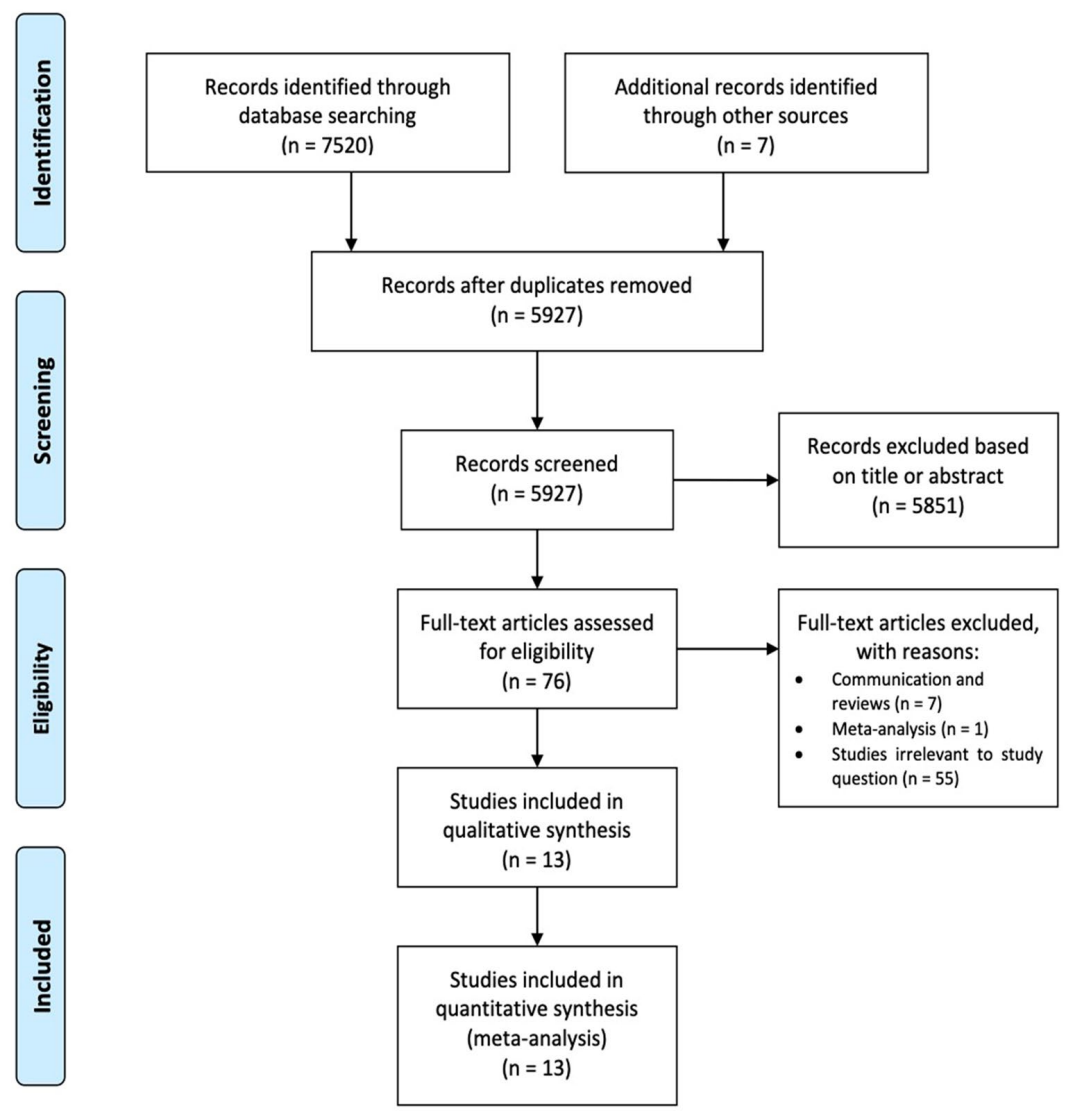
Preferred reporting items for systematic reviews and meta-analyses flow diagram.

A total of 7520 records were identified through database searching. After duplicate records were removed, 5927 citations were retrieved and their titles and abstracts were screened; 13 studies were included in the final analysis, with a total of 48,891 patients. The full PRISMA flow diagram outlining the study selection process is available in Fig. 1^7,26,27^. A complete list of studies included in the final analysis is presented in Table 1.

All studies were observational: 5 of them were unadjusted and 8 adjusted (adjustment details are shown in Table 1). Ten studies were multicenter; 3 originated from the United States, 2 from Israel, 1 each from Netherlands, Australia, Germany, Saudi Arabia, Poland, South Korea, Portugal and China.

The number of patients in each study ranged from 450 to 15,281. The mean age ranged from 56.1 to 75 years. The percentage of female sex in each study ranged from 18.0 to 43.5%. The prevalence of hypertension ranged from 43.0 to 87.3%, diabetes from 22.0 to 100.0%, smoking from 5.0 to 74.0%, prior cerebrovascular accident from 2.1 to 11.9% and prior myocardial infarction from 21.3 to 100.0%. The percentage of 1-vessel disease ranged from 0.0 to 47.2%, 2-vessels from 9.3 to 67.0% and 3-vesels from 16.3 to 100.0% (Supplementary Table 4).

### Primary outcome

**Figure 2:**
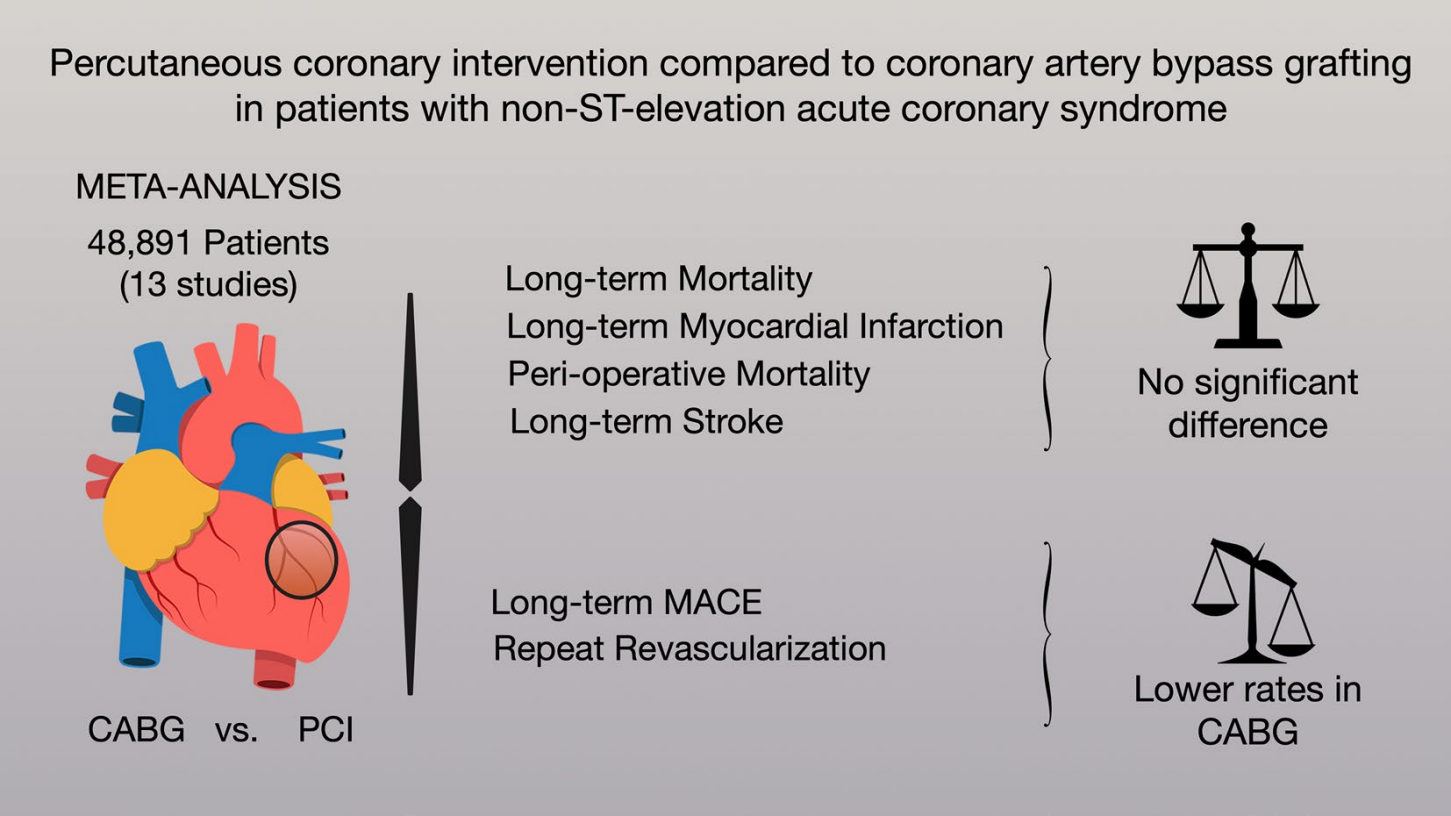
Outcomes of CABG compared with PCI in non-ST-elevation acute coronary syndrome.

Detailed results of the meta-analysis are outlined in Fig. 2 and summarized in Table 2.

**Table 2:** Outcomes summary.

Outcome	Studies no.	Patients no.	Random effect estimate (95% CI), *p*-value	Fixed effect estimate (95% CI), *p*-value	Heterogeneity (I^2^, *p*-value)
Long-term mortality	12	26,725	IRR 0.93 [0.70; 1.23], *p* = 0.61	IRR 0.80 [0.75; 0.85], *p* < 0.001	88.1%, *p* < 0.001
Long-term MACE	8	24,519	IRR 0.64 [0.54; 0.76], *p* < 0.001	IRR 0.69 [0.65; 0.73], *p* < 0.001	77.4%, *p* < 0.001
Long-term re-revascularization	6	22,573	IRR 0.37 [0.30; 0.47], *p* < 0.001	IRR 0.37 [0.30; 0.47], *p* < 0.001	42.7%, *p* = 0.11
Long-term myocardial infarction	7	7572	IRR 0.96 [0.50; 1.84], *p* = 0.61	IRR 1.56 [1.41; 1.74], *p* < 0.001	93.0%, *p* < 0.001
Peri-operative mortality	5	4334	OR 1.36 [0.94; 1.95], *p* = 0.10	OR 1.36 [0.94; 1.95], *p* = 0.10	60.0%, *p* = 0.83
Long-term stroke	6	13,197	IRR 0.96 [0.72; 1.28], *p* = 0.81	IRR 0.96 [0.72; 1.28], *p* = 0.81	0.0%, *p* = 0.497
Late MACE plus stroke	5	6842	IRR 0.67 [0.50; 0.90], *p* = 0.007	IRR 0.72 [0.66; 0.79], *p* < 0.001	86.9%, *p* < 0.001

*CI* confidence interval, *IRR* incidence rate ratio, *MACE* major adverse cardiovascular events, *No* number, *OR* odds ratios.

**Figure 3:**
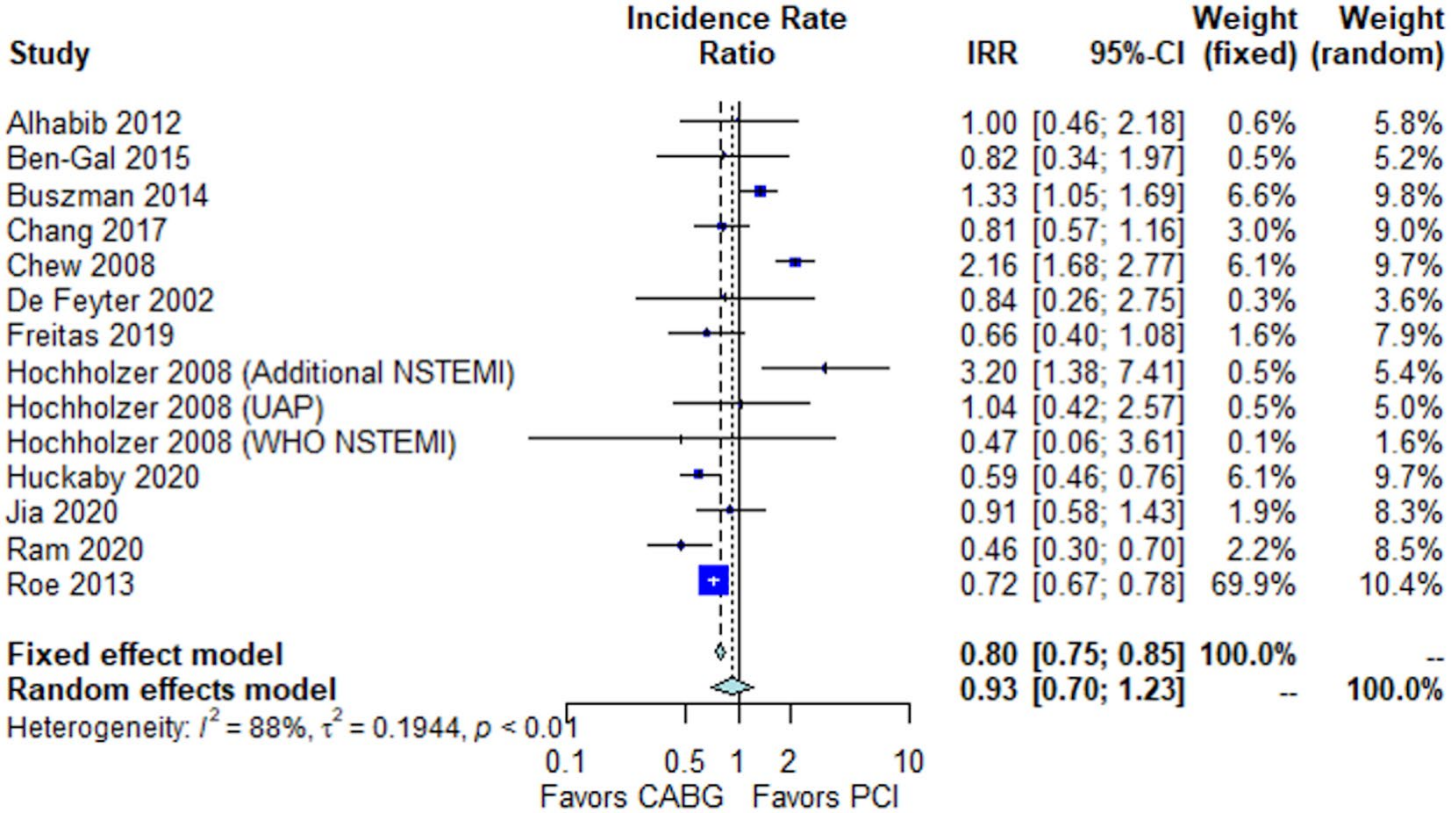
Forest plot showing pooled rates of long-term mortality in patients with non-ST-elevation acute coronary syndrome (NSTE-ACS) treated with coronary artery bypass grafting (CABG) or percutaneous coronary intervention (PCI). Abbreviations: *CABG *coronary artery bypass grafting, *CI *confidence interval, IRR incidence rate ratio, *NSTEMI *non-ST-elevation myocardial infarction, *PCI *percutaneous coronary intervention, *UAP *unstable angina pectoris, *WHO *world health organization.

No significant difference between the groups was found in long-term mortality (IRR 0.93, 95% CI 0.70; 1.23, *p* = 0.83, Fig. 3). This finding was confirmed in the sensitivity analysis comparing adjusted and low risk of bias studies vs. unadjusted studies (*p*-interaction = 0.58, Supplementary Fig. 1).

Leave-one-out analysis confirmed the solidity of the pooled estimate (Supplementary Fig. 2). Funnel plot did not demonstrate evidence of publication bias (Supplementary Fig. 3).

### Secondary outcomes

**Figure 4:**
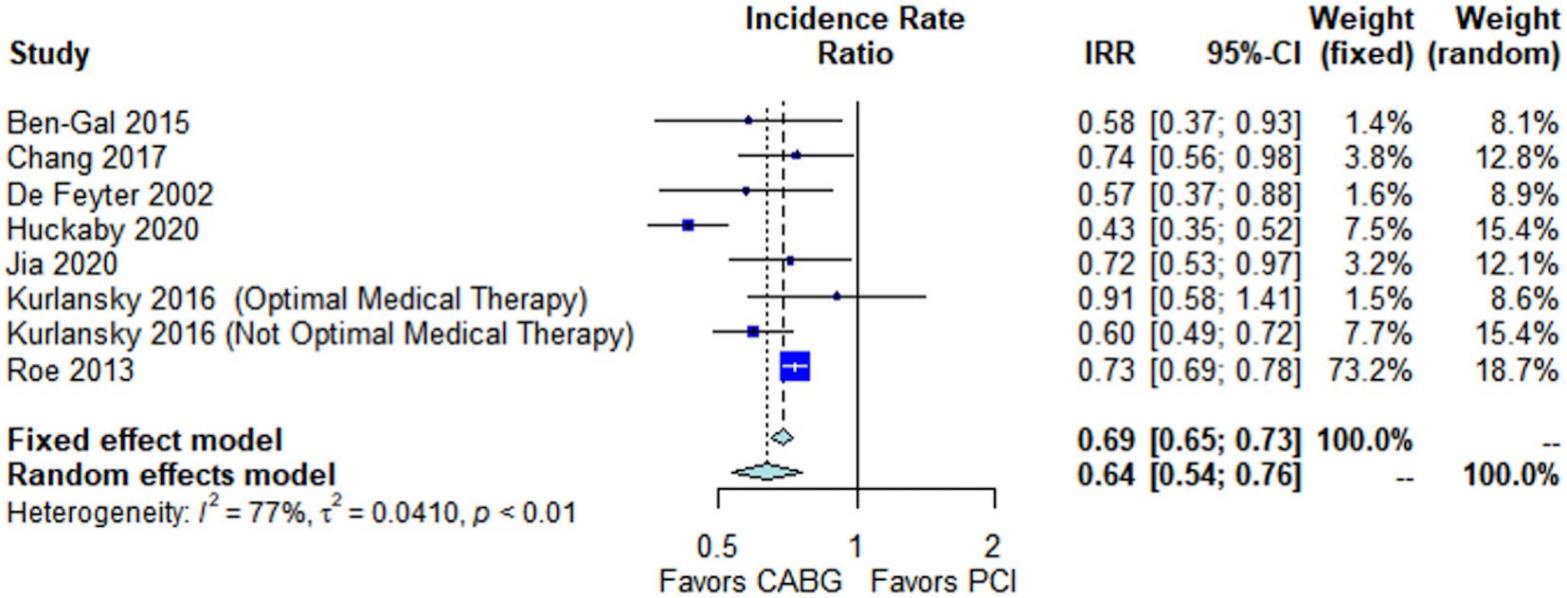
Forest plot showing pooled rates of long-term major adverse cardiovascular events (MACE) in patients with non-ST-elevation acute coronary syndrome (NSTE-ACS) treated with coronary artery bypass grafting (CABG) or percutaneous coronary intervention (PCI). Abbreviations: *CABG *coronary artery bypass grafting, *CI *confidence interval, IRR incidence rate ratio, *PCI *percutaneous coronary intervention.

**Figure 5:**
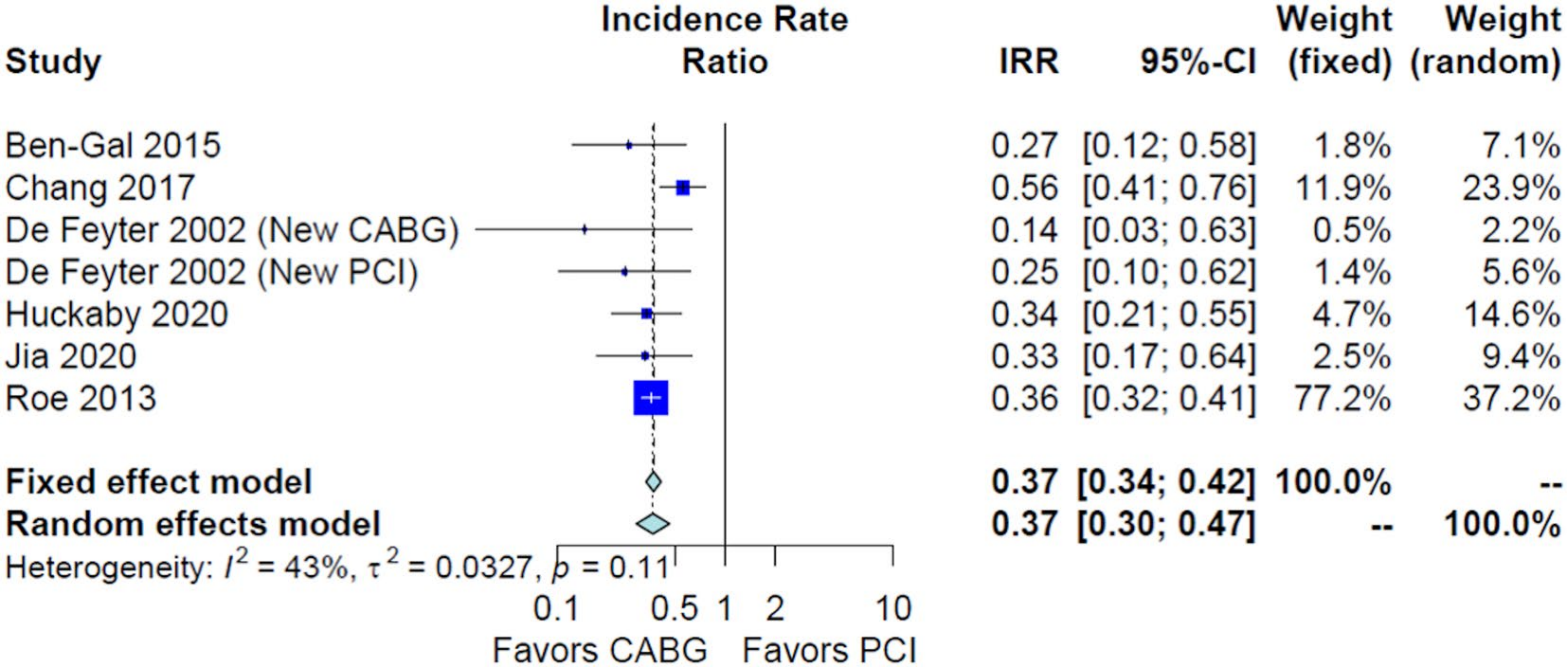
Forest plot showing pooled rates of long-term re-revascularization in patients with non-ST-elevation acute coronary syndrome (NSTE-ACS) treated with coronary artery bypass grafting (CABG) or percutaneous coronary intervention (PCI). Abbreviations: *CABG *coronary artery bypass grafting, *CI *confidence interval, IRR incidence rate ratio, *PCI *percutaneous coronary intervention.

CABG was associated with lower long-term major adverse cardiovascular events (IRR 0.64, 95% CI 0.54; 0.76, *p* < 0.001, Fig. 4) and long-term re-revascularization (IRR 0.37, 95% CI 0.30; 0.47, *p* < 0.001, Fig. 5). No significant difference was found in long-term myocardial infarction (IRR 0.96, 95% CI 0.50; 1.84, *p* = 0.61, Supplementary Fig. 4), peri-operative mortality (OR 1.36, 95% CI 0.94; 1.95, *p* = 0.10 Supplementary Fig. 5) and long-term stroke (IRR 0.96, 95% CI 0.72; 1.28, *p* = 0.81 Supplementary Fig. 6).

### Meta-regression

At meta-regression, the year of publication, the proportion of patients with prior PCI and the left main disease were inversely associated with the IRR for the primary outcome (beta = − 0.07, *p* = 0.01; beta = − 0.05, *p* = 0.004; and beta = 0.004 respectively). The proportion of 1-vessel disease was associated with the IRR for the primary outcome (beta = 0.01, *p* = 0.04, Supplementary Table 5). That means that recent studies, studies with higher percentage of patients with prior PCI and left main disease reported lower long-term mortality in the CABG group; and studies with higher percentage of patients with 1-vessel disease reported lower long-term mortality in the PCI group.

## Discussion

Our analysis suggests that for the treatment of NSTE-ACS, CABG and PCI are similar with respect to long-term mortality and myocardial infarction rates. The analysis further suggests that CABG is associated with decreased rates of long-term MACE and re-revascularization.

This is the first meta-analysis to address this important topic; our results are relevant as a substantial number of patients worldwide present with NSTE-ACS every year; in Germany over a period of 10 years 2.77 million cases of NSTE-ACS were recorded^1^. In the United States there are more than 1 million hospitalizations per year due to acute coronary syndrome^28^, with the proportion of patients with non-ST-elevation myocardial infarction being over 50% of all infarctions and increasing over time^2^. In other studies the annual incidence of NSTE-ACS has been reported with 88 per 100,000 inhabitants^29^.

Previous studies have suggested that an invasive strategy might be superior to a conservative one^3,30^, but have not summarized a recommendation for a specific invasive therapy (PCI or CABG). Currently, only 4–10% of the patients with NSTE-ACS receive CABG and 30–40% of them PCI^1,31^.

Our findings support the current guidelines, where no clear recommendation for PCI or CABG is given and a suggestion that the criteria applied for patients with stable coronary artery disease should be applied to stabilized patients with NSTE-ACS is made^4^. Both American and European guidelines recommend a heart-team approach to revascularization decisions in NSTE-ACS^4,32^ and that, factors such as extent and complexity of the coronary artery disease, as well as other factors should be considered.

It has been proposed that the survival benefit of PCI or CABG may be primarily due to an infarct-preventing mechanism rather than to revascularization per se^5,33^. This theory might provide a mechanistical explanation of our results, showing no mortality difference in 2 invasive treatments treating the same acute events. This line of argumentation finds supports in a meta-analysis we performed demonstrating an association of a survival advantage of CABG over PCI only in cases when there was also a difference in the rate of myocardial infarction^34^. This also highlights the importance of focusing on anatomically defined coronary artery disease, as recently published randomized data demonstrates that in patients with three-vessel coronary artery disease, the incidence of death, myocardial infarction, or stroke at one year was significantly lower for CABG compared to fractional flow reserve guided PCI^35^.

This argumentation is also supported by the results of the meta-regression we performed, showing that the recent studies and studies with higher percentage of patients with prior PCI reported lower long-term mortality in the CABG group; and studies with higher percentage of patients with 1-vessel disease reported lower long-term mortality in the PCI group. This seems to explain also the apparent similarity in effect estimates stemming from unadjusted and adjusted studies, with older studies being often unadjusted and the recent ones adjusted.

If we further explore the apparent discrepancies in adjusted effect estimates from some specific studies, older studies (e.g., Hochholzer et al., 2008) showed a survival advantage for PCI, while newer ones (e.g., Ram et al., 2020) showed a survival advantage for CABG. However, the percentage of the patients who received optimal medical therapy (antiplatelet, diuretic and statin therapy) was massively lower in for the CABG group in the cohort analyzed by Hochholzer (e.g., 90% vs. 2.9% for dual antiplatelet therapy), potentially providing an explanation for the observed differences.

### Study strength and limitations

This is the first meta-analysis to address this important topic. Moreover, we analyzed 5 different outcomes and performed different subgroup analyses and a meta-regression of 14 different pre-operative factors. However, this work has the intrinsic limitations of observational series, including the risk of methodological heterogeneity of the included studies, residual confounders and ecological fallacy of meta-regression. In addition, treatment allocation bias is likely present in all observational series comparing two interventions with different operative risk and invasiveness.

## Conclusion

Our analysis suggests that for the treatment of NSTE-ACS, CABG and PCI are similar with respect to long-term mortality and myocardial infarction rates. The analysis further suggests that CABG is associated with decreased rates of long-term MACE and re-revascularization. Randomized comparisons in this specific setting are necessary.

## Supplementary Information

Supplementary Information.

## Data Availability

The datasets generated analyzed during the current study are available from the corresponding author on reasonable request.

## Abbreviations

CABG: Coronary artery bypass grafting
CAD: Coronary artery disease
MACE: Major cardiovascular adverse events
MI: Myocardial infarction
NSTE-ACS: Non-ST-elevation acute coronary syndrome
NSTEMI: Non-ST-elevation myocardial infarction
PCI: Percutaneous coronary intervention
UAP: Unstable angina pectoris
