# Supplementary material for: A systematic review and meta-analysis of percutaneous coronary intervention compared to coronary artery bypass grafting in non-ST-elevation acute coronary syndrome
Source: Sci Rep. 2022 Mar 24;12:5138. doi: 10.1038/s41598-022-09158-0 (PMC8948200; doi:10.1038/s41598-022-09158-0)
Supplement: Supplementary file 1 — Supplementary Information. [file 41598_2022_9158_MOESM1_ESM.docx]

**SUPPLEMENTARY MATERIAL**

Supplementary Table 1. Search strategy for Ovid MEDLINE.

| **Line #** | **Search** | **# of results** |
| --- | --- | --- |
| 1 | **Percutaneous Coronary Intervention/** | **18879** |
| 2 | (percutaneous coronary intervention* or percutaneous coronary revasculari*ation* or percutaneous coronary angioplast* or PCI).tw. | 42336 |
| 3 | **Stents/ or Drug-Eluting Stents/ or Self Expandable Metallic Stents/** | **78404** |
| 4 | (stent or stents or stenting or stented).tw | 100822 |
| 5 | **Angioplasty, Balloon, Coronary/** | **35443** |
| 6 | (coronary balloon angioplast* or transluminal coronary balloon dilation or coronary artery balloon dilation or percutaneous transluminal coronary angioplast* or PTCA).tw. | 9805 |
| 7 | **Atherectomy, Coronary/** | **1820** |
| 8 | (coronary atherectom* or rotational atherectom*).tw | 1645 |
| 9 | or/1-8 | 166386 |
| 10 | **Coronary Artery Bypass/** | **49385** |
| 11 | **Coronary Artery Bypass, Off-Pump/** | **3342** |
| 12 | (coronary adj2 (bypass* or graft* or surger*)).tw. | 52207 |
| 13 | (CABG or aorticocoronary anastomosis or total arterial revasculari*ation* or multiple arterial revasculari*ation*).tw. | 18275 |
| 14 | **Internal Mammary-Coronary Artery Anastomosis/** or ((right internal mammary artery or RIMA or left internal mammary artery or LIMA or Coronary Internal Mammary Artery or arteria mammaria interna or arteria thoracica interna or internal thoracic artery or mammary internal artery) and (transplant* or graft* or anastomosis)).tw. | **5570** |
| 15 | (surgical revasculari*ation* or cardiac muscle revasculari*ation* or coronary revasculari*ation* or heart muscle revasculari*ation* or heart myocardium revasculari*ation* or heart revasculari*ation* or internal mammary arterial anastomosis or internal mammary arterial implant* or internal mammary artery anastomosis or internal mammary artery graft* or internal mammary artery implant* or internal mammary-coronary artery anastomosis).tw. | 11758 |
| 16 | or/1-15 | 80191 |
| 17 | (NSTE-ACS or non-ST elevat* acute coronary syndrome* or non-ST segment elevat* acute coronary syndrome* or NSTE acute coronary syndrome*).tw. | 2474 |
| 18 | Non-ST Elevated Myocardial Infarction/ | 904 |
| 19 | (NSTEMI or non-ST elevat* myocardial infarction* or NSTE myocardial infarction* or non-ST elevat* MI or non-ST elevat* heart infarction* or NSTE heart infarction* or non-STEMI).tw. | 3856 |
| 20 | Angina, Unstable/ | 9082 |
| 21 | (unstable angina* or "angina at rest" or preinfarction angina* or myocardial preinfarction syndrome*).tw. | 13574 |
| 22 | or/17-21 | 21833 |
| 23 | 9 and 16 and 22 | 1878 |
| 24 | limit 23 to English language | 1577 |

**Ovid MEDLINE** (ALL – 1946 - October 16, 2020)

Searched on October 19, 2020

Limited to English language articles

No publication date or article type restrictions

Supplementary Table 2. Newcastle-Ottawa assessing quality scale.

| **STUDY** | | **SELECTION** | **COMPARABILITY** | **OUTCOME/EXPOSURE** |
| --- | --- | --- | --- | --- |
| DE FEYTER, 2002 ^1^ | **** | | ** | *** |
| CHEW, 2008 ^2^ | **** | |  | *** |
| HOCHHOLZER, 2008 ^3^ | **** | |  | *** |
| ALHABIB, 2012 ^4^ | **** | | * | *** |
| ROE, 2013 ^5^ | *** | | * | *** |
| BUSZMAN, 2014 ^6^ | **** | | ** | *** |
| BEN-GAL, 2015 ^7^ | *** | | ** | *** |
| KURLANSKY, 2016 ^8^ | **** | | ** | *** |
| CHANG, 2017 ^9^ | **** | | ** | *** |
| FREITAS, 2019 ^10^ | **** | | ** | *** |
| HUCKABY, 2020 ^11^ | *** | | * | *** |
| JIA, 2020 ^12^ | *** | | ** | *** |
| RAM, 2020 ^13^ | **** | | ** | *** |

The stars correspond the given points.

Supplementary Table 3. Definition for reported major adverse cardiovascular events (MACE) and stroke endpoints.

| **STUDY** | **MACE DEFINITION** | **STROKE DEFINITION** |
| --- | --- | --- |
| DE FEYTER, 2002 ^1^ | ALL-CAUSE MORTALITY, CARDIOVASCULAR ACIDENT, DOCUMENTAED NONFATAL MYOCARDIAL INFARCTION OR REPEAT REVASCULARIZATION | CEREBROVASCULAR EVENTS: STROKE, TRANSIENT ISCHEMIC ATTACKS, AND REVERSIBLE ISCHEMIC NEUROLOGICAL DEFICITS |
| CHEW, 2008 | NOT REPORTED | ANY SPECIFIC DEFINITION |
| ROE, 2013 ^5^ | ALL-CAUSE MORTALITY, READMISSION FOR MI OR READMISSION FOR STROKE | NOT REPORTED |
| BEN-GAL, 2015 ^7^ | ALL-CAUSE MORTALITY, MYOCARDIAL INFARCTION OR UNPLANNED REVASCULARIZATION | ANY SPECIFIC DEFINITION |
| KURLANSKY, 2016 ^8^ | ALL-CAUSE MORTALITY, NON-FATAL MYOCARDIAL INFARCTION OR REPEAT REVASCULARIZATION PROCEDURE | NOT REPORTED |
| CHANG, 2017 ^9^ | ALL-CAUSE MORTALITY, MYOCARDIAL INFARCTION OR STROKE | ANY SPECIFIC DEFINITION |
| HUCKABY, 2020 ^11^ | ALL-CAUSE MORTALITY, MYOCARDIAL INFARCTION, STROKE OR THE NEED FOR ANY REPEAT REVASCULARIZATION | ANY SPECIFIC DEFINITION |
| JIA, 2020 ^12^ | ALL-CAUSE MORTALITY, MYOCARDIAL INFARCTION, STROKE OR UNPLANNED REVASCULARIZATION | ANY SPECIFIC DEFINITION |

Supplementary Table 4. Demographics of the patients included (part 1).

|  | **AGE (MEAN±SD)** |  | **FEM (%)** |  | **MEAN LVEF  (MEAN±SD)** |  | **HP (%)** |  | **DM (%)** |  | **SMOKING (%)** |  | **PRIOR CVA (%)** |  | **PRIOR MI (%)** |  | **PRIOR PCI (%)** |  |
| --- | --- | --- | --- | --- | --- | --- | --- | --- | --- | --- | --- | --- | --- | --- | --- | --- | --- | --- |
|  |  |  |  |  |  |  |  |  |  |  |  |  |  |  |  |  |  |  |
| **STUDY** | **CABG** | **PCI** | **CABG** | **PCI** | **CABG** | **PCI** | **CABG** | **PCI** | **CABG** | **PCI** | **CABG** | **PCI** | **CABG** | **PCI** | **CABG** | **PCI** | **CABG** | **PCI** |
| **DE FEYTER, 2002 ^1^** | 61±10 | 61±10 | 25 | 20 | 59±14 | 62±13 | 43 | 49 | 15 | 19 | 71 | 74 | NR | NR | 48 | 46 | NR | NR |
| **CHEW, 2008 ^2^** | 68 | NR | 25.7 | NR | NR | NR | 69.4 | NR | 33.2 | NR | 24.1 | NR | 5.2 | NR | 46 | NR | 14.6 | NR |
| **HOCHHOLZER, 2008 (ADDITIONAL NSTEMI) ^3^** | 69.3 | 68.8 | 18 | 27 | NR | NR | 71 | 68 | 26 | 22 | 16 | 29 | NR | NR | 43 | 38 | 10 | 13 |
| **HOCHHOLZER, 2008 (WHO NSTEMI) ^3^** | 69.6 | 62.2 | 23 | 24 | NR | NR | 56 | 63 | 36 | 15 | 5 | 40 | NR | NR | 41 | 26 | 5 | 15 |
| **HOCHHOLZER, 2008 (UAP) ^3^** | NR | NR | NR | NR | NR | NR | NR | NR | NR | NR | NR | NR | NR | NR | NR | NR | NR | NR |
| **ALHABIB, 2012 ^4^** | 59.7±11.2 | 56.1±11.5 | 19.5 | 19.1 | NR | NR | 56.7 | 57.4 | 58.6 | 45.6 | 31.7 | 35.7 | 3.7 | 2.1 | 26.8 | 28.7 | 8.6 | 17.1 |
| **ROE, 2013 ^5^** | 74 | 75 | 34.3 | 43.5 | NR | NR | 74.7 | 75.7 | 33.9 | 32.3 | 15.3 | 13.9 | 8.3 | 9.6 | 21.3 | 27.4 | 15.1 | 26.2 |
| **BUSZMAN, 2014 ^6^** | 65.2±9.9 | 65.3±9.9 | 29.7 | 28.3 | 51.6±11.2 | 51.8±10.2 | 83.7 | 84.3 | 28.4 | 28.8 | 40.9 | 43.3 | NR | NR | 30.4 | 30.1 | 8.1 | 7.8 |
| **BEN-GAL, 2015 ^7^** | 65 | 65 | 30.1 | 32.5 | 50 | 53 | 82.2 | 81 | 100 | 100 | 20.6 | 29.2 | NR | NR | 28.2 | 33.1 | 31.3 | 31.3 |
| **KURLANSKY, 2016 ^8^** | 64.8±10.3 | 65.3±11.9 | 30.3 | 31.8 | 50.1±11.9 | 53.8±16.8 | 79.2 | 76 | 34.4 | 33.9 | 52.7 | 44.5 | 8 | 4.7 | 31.1 | 27.6 | 18.6 | 35.2 |
| **CHANG, 2017 ^9^** | 65.1±9.8 | 64.1±10.2 | 26.7 | 30.2 | NR | NR | 60.4 | 61.4 | 32.6 | 36.8 | 24.9 | 22.5 | 5.6 | 6.2 | 22.9 | 22.2 | NR | NR |
| **FREITAS, 2019 ^10^** | 68 | 66 | 27 | 30 | NR | NR | 76 | 72 | 57 | 59 | 21 | 25 | 11.9 | 8.8 | 24 | 24 | 17 | 14 |
| **HUCKABY, 2020 ^11^** | 67 | 70 | 30.1 | 36.5 | 50 | 50 | 87.3 | 83.7 | 49.5 | 47.4 | 30.5 | 22.1 | NR | NR | 100 | 26.5 | 23.6 | 31.5 |
| **JIA, 2020 ^12^** | 61.7±8.7 | 61.1±10.1 | 20.5 | 24.2 | NR | NR | 71 | 71.3 | 33.7 | 33.7 | 53.7 | 54.2 | 10.6 | 9.3 | 37.5 | 31.8 | NR | NR |
| **RAM, 2020 ^13^** | 65±11 | 64±12 | 22 | 25 | NR | NR | 67 | 67 | 45 | 44 | 32 | 36 | 9 | 7 | 31 | 30 | 28 | 28 |

CABG= coronary arterial bypass graft; CVA= cerebrovascular accident; DM= diabetes; FEM= female; HP= hypertension, LVEF= left ventricular ejection fraction; MI= myocardial infarction; NR= not reported; NSTEMI= non-ST-elevation myocardial infarction, PCI= percutaneous coronary intervention; SD= standard deviation, UAP= unstable angina pectoris, WHO= world health organization.

Supplementary Table 4. Demographics of the patients included (part 2).

|  | **1-VESSEL (%)** |  | **2-VESSELS (%)** |  | **3-VESSELS (%)** |  | **USE OF DES (%)** | **TOTAL ARTERIAL (%)** | **LM DISEASE (%)** |  | **DISCHARGED WITH**  **ASA (%)** |  | **DISCHARGED WITHE**  **ADP INHIBITOR(%)** |  |
| --- | --- | --- | --- | --- | --- | --- | --- | --- | --- | --- | --- | --- | --- | --- |
| **STUDY** | **CABG** | **PCI** | **CABG** | **PCI** | **CABG** | **PCI** | **PCI** | **CABG** | **PCI** | **CABG** | **PCI** | **CABG** | **PCI** | **CABG** |
| **DE FEYTER, 2002 ^1^** | NR | NR | 64 | 67 | 36 | 33 | NR | NR | 93 | 90 | NR | NR | NR | NR |
| **CHEW, 2008 ^2^** | NR | NR | NR | NR | NR | NR | NR | NR | NR | NR | NR | NR | NR | NR |
| **HOCHHOLZER, 2008 (ADDITIONAL NSTEMI) ^3^** | NR | NR | NR | NR | NR | NR | 0 | NR | NR | NR | 96 | 94 | NR | NR |
| **HOCHHOLZER, 2008 (WHO NSTEMI) ^3^** | NR | NR | NR | NR | NR | NR | 0 | NR | NR | NR | 93 | 89 | NR | NR |
| **HOCHHOLZER, 2008 (UAP) ^3^** | NR | NR | NR | NR | NR | NR | 0 | NR | NR | NR | NR | NR | NR | NR |
| **ALHABIB, 2012 ^4^** | 2 | 47.2 | 20.4 | 32.9 | 74.8 | 16.3 | NR | NR | 1.7 | 33.3 | 97.6 | 95.7 | 96.2 | 56.7 |
| **ROE, 2013 ^5^** | 4.8 | 37 | 23.4 | 34.3 | 71.8 | 28.7 | NR | NR | NR | NR | 97.5 | 95.4 | 96.4 | 29.8 |
| **BUSZMAN, 2014 ^6^** | NR | NR | 28.3 | 30.6 | 71.7 | 69.4 | 10.2 | NR | 2.4 | 13.7 | NR | NR | NR | NR |
| **BEN-GAL, 2015 ^7^** | NR | NR | 25.4 | 20.7 | 74.6 | 79.3 | NR | NR | 5.5 | 26.7 | 100 | 100 | 100 | 100 |
| **KURLANSKY, 2016 ^8^** | NR | NR | NR | NR | 41.1 | 17.8 | NR | NR | NR | NR | NR | NR | NR | NR |
| **CHANG, 2017 ^9^** | NR | NR | 9.3 | 9.3 | 51.4 | 51.6 | 100 | NR | 3.9 | 5.2 | 97.4 | 95.2 | 96.4 | 63.6 |
| **FREITAS, 2019 ^10^** | NR | NR | NR | NR | 63 | 59 | NR | NR | 13 | 37 | NR | NR | NR | NR |
| **HUCKABY, 2020 ^11^** | 2.3 | 0 | 19.9 | 38 | 77.7 | 59.3 | NR | NR | NR | NR | NR | NR | NR | NR |
| **JIA, 2020 ^12^** | NR | NR | NR | NR | 100 | 100 | NR | NR | 12.6 | 38.1 | 98 | 93.7 | 92.9 | 8 |
| **RAM, 2020 ^13^** | 5 | 7 | 25 | 25 | 70 | 68 | NR | NR | NR | NR | NR | NR | NR | NR |

ADP= adenosine diphosphate ASA= acetylsalicylic acid, CABG= coronary arterial bypass graft; DES= drug eluting stents; LM=left main, NR= not reported; NSTEMI= non-ST-elevation myocardial infarction, PCI= percutaneous coronary intervention; SD= standard deviation, UAP= unstable angina pectoris, WHO= world health organization.

Supplementary Table 5. Meta-regression for the primary outcome.

| Variables | Beta ± SE, P value |
| --- | --- |
| Year of publication | **-0.0658 ± 0.0255, P=0.0100** |
| Mean follow-up time (years) | -0.1099 ± 0.0644, P=0.0883 |
| Age (mean) | 0.0075 ± 0.0419, P=0.8579 |
| Female (%) | -0.0274 ± 0.0286, P=0.3382 |
| Mean LVEF (%) | 0.0155 ± 0.0874, P=0.8596 |
| Hypertension (%) | -0.0016 ± 0.0154, P=0.9190 |
| Diabetes (%) | -0.0102 ± 0.0085, P=0.2297 |
| Smoking (%) | -0.0005 ± 0.0120, P=0.9677 |
| Prior CVA (%) | -0.1042 ± 0.0721, P=0.1482 |
| Prior MI (%) | 0.0063 ± 0.0139, P=0.6499 |
| Prior PCI (%) | **-0.0506 ± 0.0178, P=0.0044** |
| 1-vessel (%) | **0.0144 ± 0.0072, P=0.0439** |
| 2-vessel (%) | 0.0022 ± 0.0128, P=0.8642 |
| 3-vessel (%) | 0.0018 ± 0.0076, P=0.8135 |
| Left main disease | **-0.0044 ± 0.0073, P=0.0100** |
| Use of DES | -0.0063 ± 0.0046, P=0.1735 |
| Discharged with ASA | -0.0628 ± 0.1089, P=0.5641 |
| Discharged with ADP inhibitor | 0.0024 ± 0.0072, P=0.7358 |

Statistically significant variables are highlighted. ASA= acetylsalicylic acid,
ADP= Adenosine diphosphate, CVA= cerebrovascular accident; LVEF= left ventricular ejection fraction; MI= myocardial infarction; PCI= percutaneous coronary intervention; SE= standard error

Supplementary Table 6. Unstable angina pectoris and non-ST-elevation myocardial infarction definition.

| **STUDY** | **PATIENTS SYNDROM INCLUDED** | **SYNDROM DEFINITION/SOURCE** |
| --- | --- | --- |
| DE FEYTER, 2002 ^1^ | UNSTABLE ANGINA | Braunwald classification (I B, C through III B, C) |
| CHEW, 2008 ^2^ | UNSTABLE ANGINA AND NSTEMI | Ischemic pain and at least 2 of the following: a rise in troponin or CK-MB above the upper limit of normal; new or presumed new ST-segment depression or transient ST elevation; age ≥60 years |
| HOCHHOLZER, 2008 ^3^ | UNSTABLE ANGINA AND NSTEMI | World Health Organization (WHO) definition and European Society of Cardiology/American College of Cardiology |
| ALHABIB, 2012 ^4^ | UNSTABLE ANGINA AND NSTEMI | American College of Cardiology |
| ROE, 2013 ^5^ | NSTEMI | Ischemic chest pain lasting ≥10 minutes within the previous 24 hours and were found to have either positive local laboratory cardiac markers (either creatine kinase-MB or troponin levels above the upper limit of normal) |
| BUSZMAN, 2014 ^6^ | UNSTABLE ANGINA AND NSTEMI | European Society of Cardiology |
| BEN-GAL, 2015 ^7^ | UNSTABLE ANGINA AND NSTEMI | Enrolled patients were aged ≥18 years with symptoms of ACS lasting ≥10 minutes within the preceding 24 hours and with ≥1 of the following criteria: new ST-segment depression or transient el- evation ≥1 mm; elevated troponin I, troponin T, or creatine kinase- MB; known coronary artery disease; or the presence of all 4 other thrombolysis in myocardial infarction risk criteria |
| KURLANSKY, 2016 ^8^ | NSTEMI | Not specified |
| CHANG, 2017 ^9^ | UNSTABLE ANGINA AND NSTEMI | Defiined in by the individual trials |
| FREITAS, 2019 ^10^ | NSTEMI | American College of Cardiology |
| HUCKABY, 2020 ^11^ | NSTEMI | Cardiac biomarkers (ie, troponin, creatine kinase‐myocardial band) exceeding the upper limit of normal with a clinical presentation consistent with or suggestive of cardiac ischemia and absence of electrocardiogram changes diagnostic of a STEMI. |
| JIA, 2020 ^12^ | UNSTABLE ANGINA AND NSTEMI | European Society of Cardiology/American College of Cardiology |
| RAM, 2020 ^13^ | UNSTABLE ANGINA AND NSTEMI | American College of Cardiology/American Heart Association |

NSTEMI: non-ST-elevation myocardial infarction.

| **STUDY** | **USE OF DRUG ELUTING STENT (%)** |
| --- | --- |
| DE FEYTER, 2002 ^1^ | Not Reported |
| CHEW, 2008 ^2^ | Not Reported |
| HOCHHOLZER, 2008 (ADDITIONAL NSTEMI) ^3^ | 0 |
| HOCHHOLZER, 2008 (WHO NSTEMI) ^3^ | 0 |
| HOCHHOLZER, 2008 (UAP) ^3^ | 0 |
| ALHABIB, 2012 ^4^ | Not Reported |
| ROE, 2013 ^5^ | Not Reported |
| BUSZMAN, 2014 ^6^ | 10.2 |
| BEN-GAL, 2015 ^7^ | Not Reported |
| KURLANSKY, 2016 ^8^ | Not Reported |
| CHANG, 2017 ^9^ | 100 |
| FREITAS, 2019 ^10^ | Not Reported |
| HUCKABY, 2020 ^11^ | Not Reported |
| JIA, 2020 ^12^ | Not Reported |
| RAM, 2020 ^13^ | Not Reported |

Supplementary Table 7. Use of drug eluting stent in the included studies.

NSTEMI= non-ST-elevation myocardial infarction, UAP= unstable angina pectoris, WHO= world health organization.

Supplementary Figure 1. Sensitivity analysis for long-term mortality based in studies with adjusted vs. unadjusted populations.


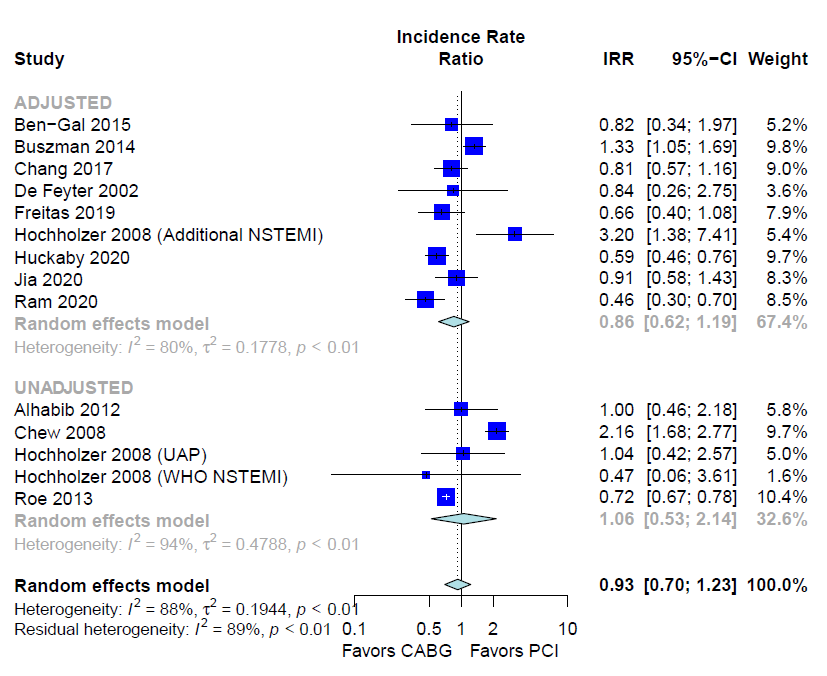


Supplementary Figure 2. Leave-one-out analysis for long-term mortality.


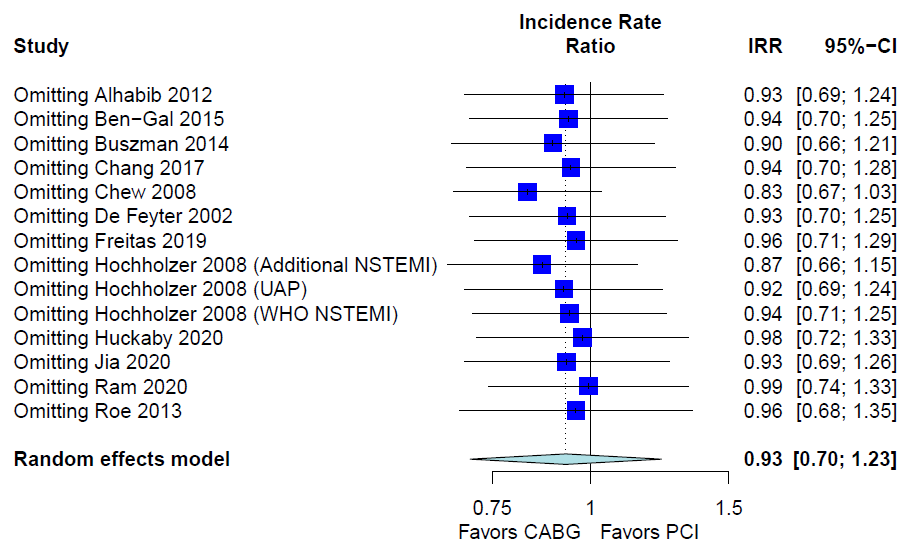


Supplementary Figure 3. Funnel plot for long-term mortality.


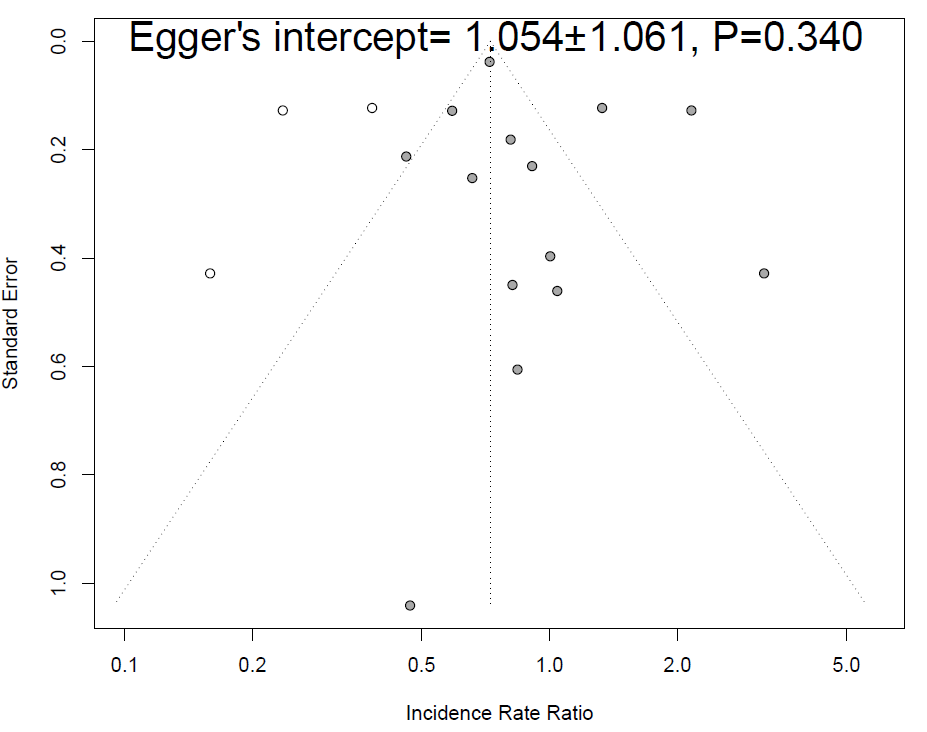


Supplementary Figure 4. Forest plot for long-term myocardial infarction.


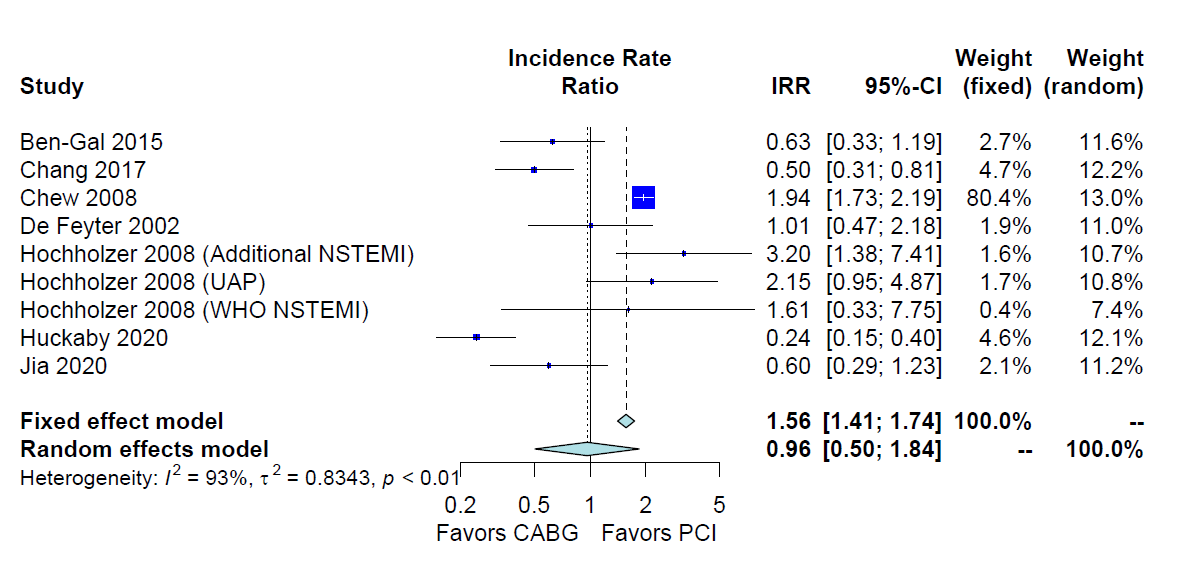


Supplementary Figure 5. Forest plot for peri-operative mortality.


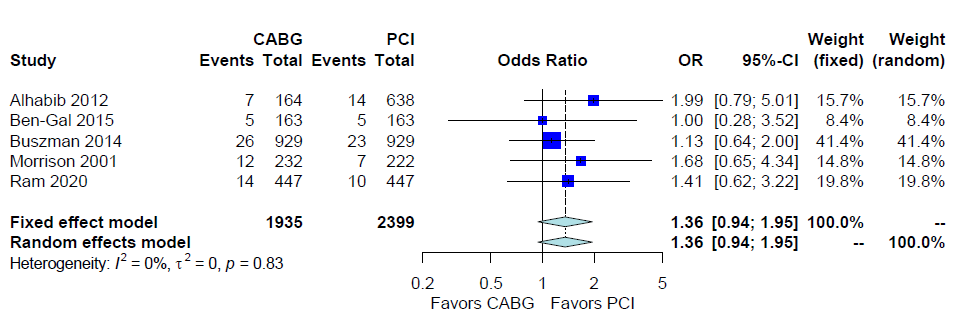


Supplementary Figure 6. Forest plot for long-term stroke.

Supplementary Figure 7. Forest plot for a cumulative meta-analysis for long-term mortality.


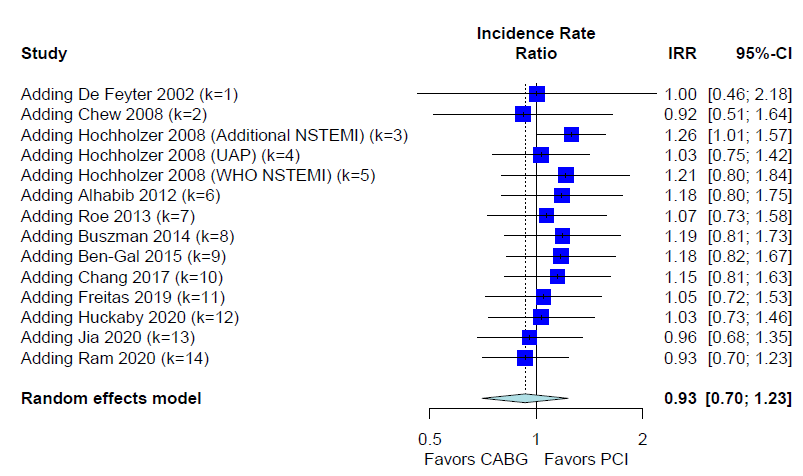


Supplementary Figure 8. Funnel plots for the assessment of publication bias for the secondary outcomes.

| Late MACE  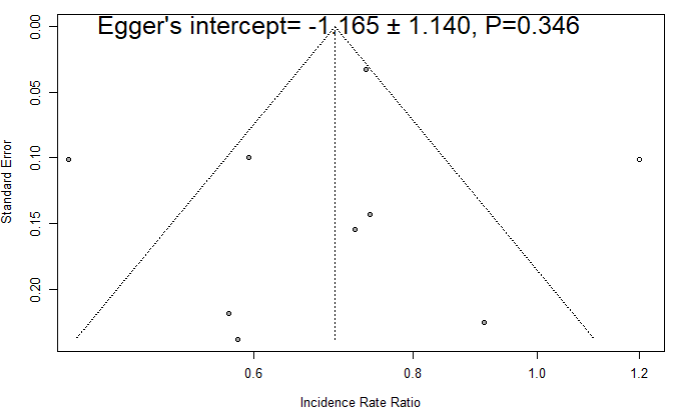 | Late re-revascularization  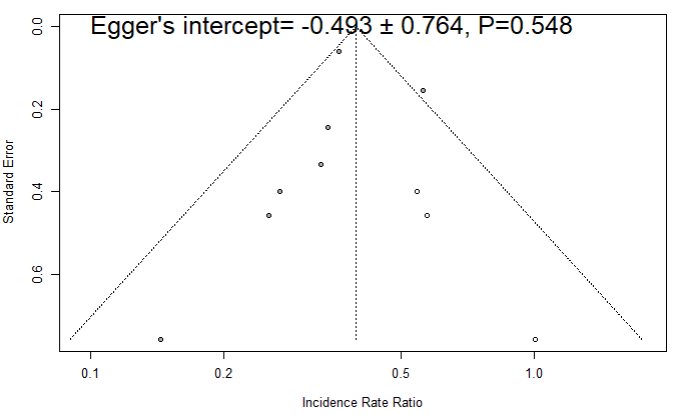 |
| --- | --- |
| Late myocardial infarction  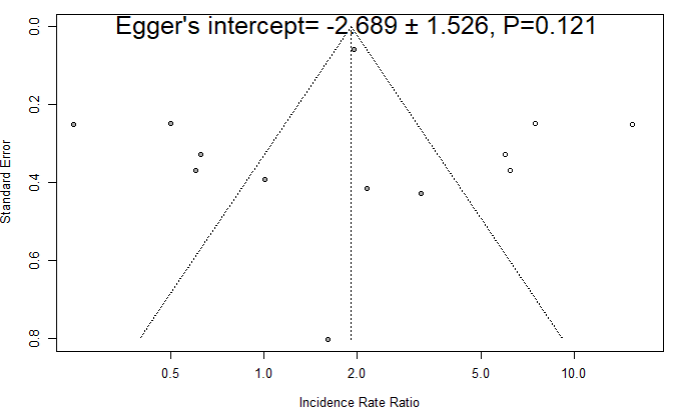 | Peri-operative mortality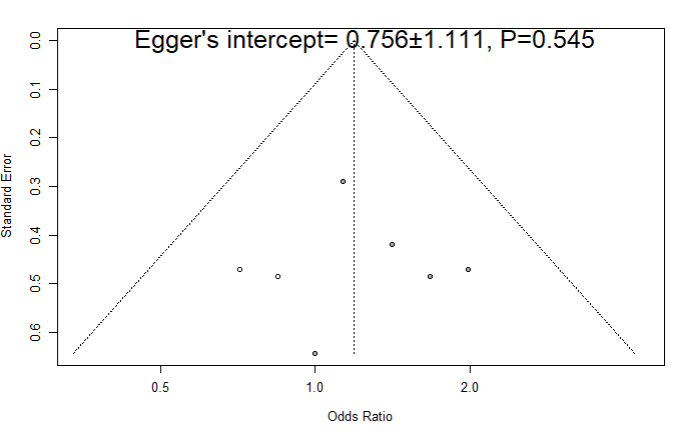 |
| Late stroke  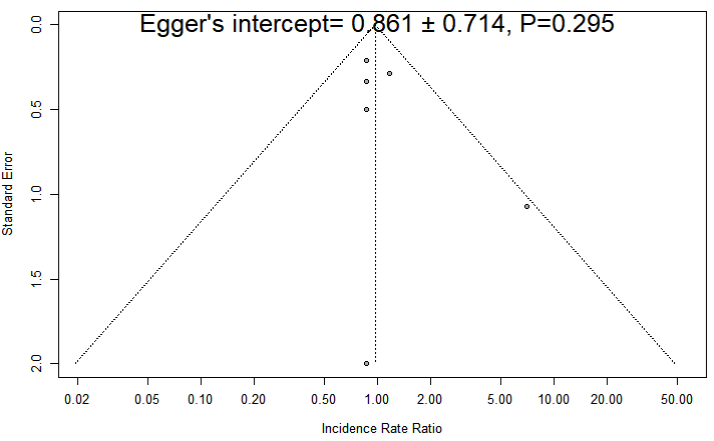 |  |

Supplementary Figure 9. Bubble plots of meta-regression of significant covariates on long-term mortality.

| 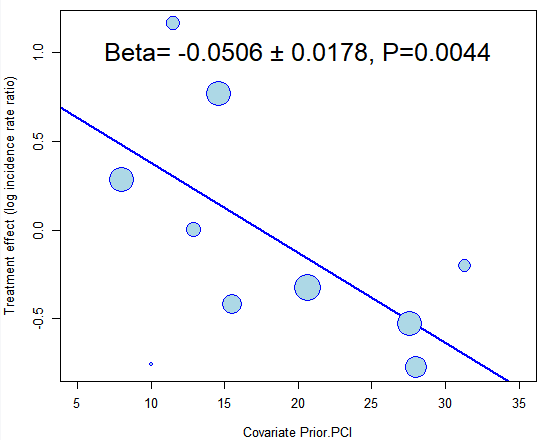 | 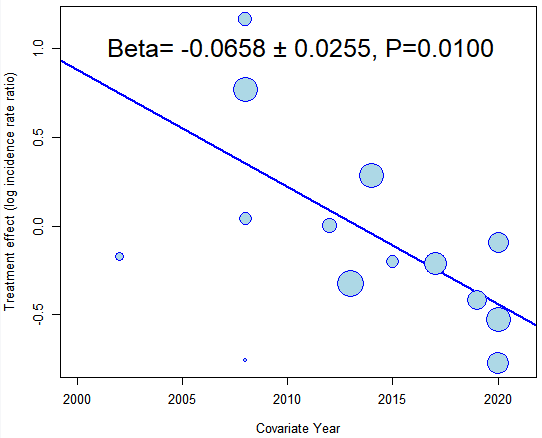 |
| --- | --- |
| 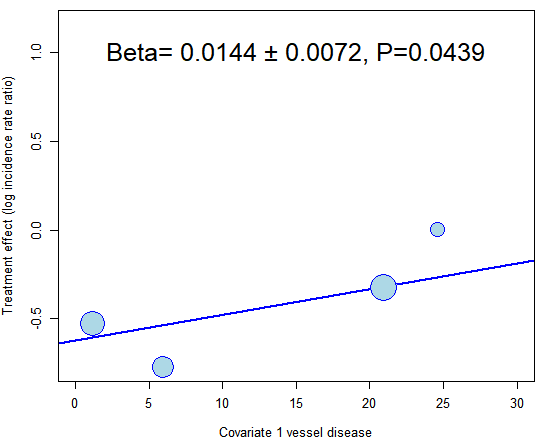 | 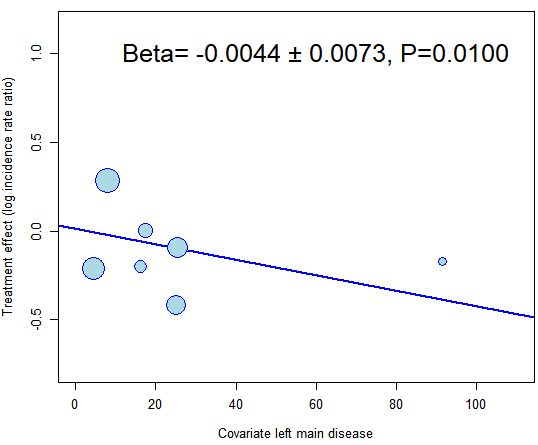 |

**FIGURE LEGENDS**

Supplementary Figure 1. Forest plot for sensitivity analysis of long-term mortality based in adjusted and low risk of bias studies vs. unadjusted populations of patients with non-ST-elevation acute coronary syndrome (NSTE-ACS) treated with coronary arterial bypass grafting (CABG) or percutaneous coronary intervention (PCI). Abbreviations: CABG= coronary arterial bypass grafting, CI= confidence interval, IRR= incidence rate ratio, NSTEMI= non-ST-elevation myocardial infarction, PCI= percutaneous coronary intervention, UAP= unstable angina pectoris, WHO= world health organization.

Supplementary Figure 2. Leave-one-out analysis for long-term mortality showing pooled rates of long-term mortality in patients with non-ST-elevation acute coronary syndrome (NSTE-ACS) treated with coronary arterial bypass grafting (CABG) or percutaneous coronary intervention (PCI). Abbreviations: CABG= coronary arterial bypass grafting, CI= confidence interval, IRR= incidence rate ratio, NSTEMI= non-ST-elevation myocardial infarction, PCI= percutaneous coronary intervention, UAP= unstable angina pectoris, WHO= world health organization.

Supplementary Figure 3. Funnel plot for the assessment of publication bias for the main outcome of peri-operative mortality. Black dots represent individual studies. Dashed lines represent the pseudo confidence interval. Visual inspection and Egger test suggest no evident asymmetry.

Supplementary Figure 4. Forest plot showing pooled rates of long-term myocardial infarction in patients with non-ST-elevation acute coronary syndrome (NSTE-ACS) treated with coronary arterial bypass grafting (CABG) or percutaneous coronary intervention (PCI). Abbreviations: CABG= coronary arterial bypass grafting, CI= confidence interval, IRR= incidence rate ratio, NSTEMI= non-ST-elevation myocardial infarction, PCI= percutaneous coronary intervention, UAP= unstable angina pectoris, WHO= world health organization.

Supplementary Figure 5. Forest plot showing pooled rates of peri-operative mortality in patients with non-ST-elevation acute coronary syndrome (NSTE-ACS) treated with coronary arterial bypass grafting (CABG) or percutaneous coronary intervention (PCI). Abbreviations: CABG= coronary arterial bypass grafting, CI= confidence interval, OR= odds ratio, PCI= percutaneous coronary intervention.

Supplementary Figure 6. Forest plot showing pooled rates of long-term stroke in patients with non-ST-elevation acute coronary syndrome (NSTE-ACS) treated with coronary arterial bypass grafting (CABG) or percutaneous coronary intervention (PCI). Abbreviations: CABG= coronary arterial bypass grafting, CI= confidence interval, OR= odds ratio, PCI= percutaneous coronary intervention.

Supplementary Figure 7. Forest plot for cumulative meta-analysis of long-term mortality of patients with non-ST-elevation acute coronary syndrome (NSTE-ACS) treated with coronary arterial bypass grafting (CABG) or percutaneous coronary intervention (PCI). Abbreviations: CABG= coronary arterial bypass grafting, CI= confidence interval, IRR= incidence rate ratio, NSTEMI= non-ST-elevation myocardial infarction, PCI= percutaneous coronary intervention, UAP= unstable angina pectoris, WHO= world health organization.

Supplementary Figure 8. Funnel plots for the assessment of publication bias for the secondary outcomes. Black dots represent individual studies. Dashed lines represent the pseudo confidence interval. Visual inspection and Egger test suggest no evident asymmetry.

Supplementary Figure 9. Bubble plots of meta-regression of significant covariates on long-term mortality. The treatment effect for each study included is plotted along the Y-axis while the meta-regression covariate is plotted along the X-axis. Each bubble size is inversely proportional to the variance and directly related to the sample size. PCI= percutaneous coronary intervention.

**BIBLIOGRAPHIC REFERENCES**

**1.** de Feyter PJ, Serruys PW, Unger F, et al. Bypass surgery versus stenting for the treatment of multivessel disease in patients with unstable angina compared with stable angina. *Circulation.* 2002;105:2367-2372.

**2.** Chew DP, Huang Z, Pieper KS, et al. Patients with non-ST-elevation acute coronary syndromes undergoing coronary artery bypass grafting in the modern era of antithrombotic therapy. *Am Heart J.* 2008;155:239-244.

**3.** Hochholzer W, Buettner HJ, Trenk D, et al. Percutaneous Coronary Intervention Versus Coronary Artery Bypass Grafting as Primary Revascularization in Patients With Acute Coronary Syndrome. *The American Journal of Cardiology.* 2008;102:173-179.

**4.** AlHabib KF, Hersi A, Alsheikh-Ali AA, et al. Prevalence, predictors, and outcomes of conservative medical management in non-ST-segment elevation acute coronary syndromes in Gulf RACE-2. *Angiology.* 2012;63:109-118.

**5.** Roe MT, Li S, Thomas L, et al. Long-term outcomes after invasive management for older patients with non-ST-segment elevation myocardial infarction. *Circ Cardiovasc Qual Outcomes.* 2013;6:323-332.

**6.** Buszman PE, Buszman PP, Bochenek A, et al. Comparison of Stenting and Surgical Revascularization Strategy in Non-ST Elevation Acute Coronary Syndromes and Complex Coronary Artery Disease (from the Milestone Registry). *American Journal of Cardiology.* 2014;114:979-987.

**7.** Ben-Gal Y, Mohr R, Feit F, et al. Surgical versus percutaneous coronary revascularization for multivessel disease in diabetic patients with non-ST-segment-elevation acute coronary syndrome: analysis from the Acute Catheterization and Early Intervention Triage Strategy trial. *Circ Cardiovasc Interv.* 2015;8.

**8.** Kurlansky P, Herbert M, Prince S, Mack M. Coronary Artery Bypass Graft Versus Percutaneous Coronary Intervention. *Circulation.* 2016;134:1238-1246.

**9.** Chang M, Lee CW, Ahn J-M, et al. Comparison of Outcome of Coronary Artery Bypass Grafting Versus Drug-Eluting Stent Implantation for Non&#x2013;ST-Elevation Acute Coronary Syndrome. *American Journal of Cardiology.* 2017;120:380-386.

**10.** Freitas P, Madeira M, Raposo L, et al. Coronary Artery Bypass Grafting Versus Percutaneous Coronary Intervention in Patients With Non&#x2013;ST-Elevation Myocardial Infarction and Left Main or Multivessel Coronary Disease. *American Journal of Cardiology.* 2019;123:717-724.

**11.** Huckaby LV, Sultan I, Mulukutla S, et al. Revascularization following non-ST elevation myocardial infarction in multivessel coronary disease. *J Card Surg.* 2020;35:1195-1201.

**12.** Jia S, Zhang C, Jiang L, et al. Comparison of Percutaneous Coronary Intervention, Coronary Artery Bypass Grafting and Medical Therapy in Non-ST Elevation Acute Coronary Syndrome Patients With 3-Vessel Disease. *Circ J.* 2020;84:1718-1727.

**13.** Ram E, Sternik L, Klempfner R, et al. Outcomes of different revascularization strategies among patients presenting with acute coronary syndromes without ST elevation. *The Journal of Thoracic and Cardiovascular Surgery.* 2020;160:926-935.e926.
